# Transient sinoatrial node dysfunction after pulsed-field pulmonary vein ablation: an image case report

**DOI:** 10.3389/fcvm.2026.1745619

**Published:** 2026-01-29

**Authors:** Enyuan Zhang, Le He, Henan Zhang, Jing Xu, Fengmin Lu, Dongyan Wu, Yitong Yin, Wei Ma

**Affiliations:** 1 Department of Graduate School of Tianjin Medical University, Tianjin Medical University, Tianjin, China; 2 Heart Rhythm Center, Department of Cardiology, Chest Hospital, Tianjin University, Tianjin, China

**Keywords:** atrial fibrillation, case report, pulmonary vein isolation, pulsed-field ablation, sinoatrial node dysfunction

## Abstract

Pulmonary vein pulsed-field ablation (PFA) is widely regarded as a safe procedure for patients with atrial fibrillation (AF), with sinoatrial disturbances as a rare complication. A 62-year-old female patient with paroxysmal AF underwent ablation using an 8-polar circular PFA catheter. During pulmonary vein isolation (PVI) of the right superior pulmonary vein, an intermittent increase in sinus rate was noted. Recurrent sinoatrial block was observed shortly after the procedure but resolved completely within six hours. This report presents the first documented case of transient sinoatrial node dysfunction as a complication of pulmonary vein PFA. Although the underlying mechanism—whether singular or multifactorial—remains unconfirmed, this case highlights the need for caution when utilizing the 8-polar circular PFA catheter.

## Introduction

Pulsed field ablation (PFA) has emerged as a promising non-thermal technique for pulmonary vein isolation (PVI) in atrial fibrillation (AF), providing myocardial tissue selectivity and a favorable safety profile by minimizing collateral damage to adjacent structures such as the esophagus and phrenic nerve (1). However, its effects on the sinoatrial (SA) node and the autonomic nervous system remain incompletely understood.

## Case presentation

**Figure 1:**
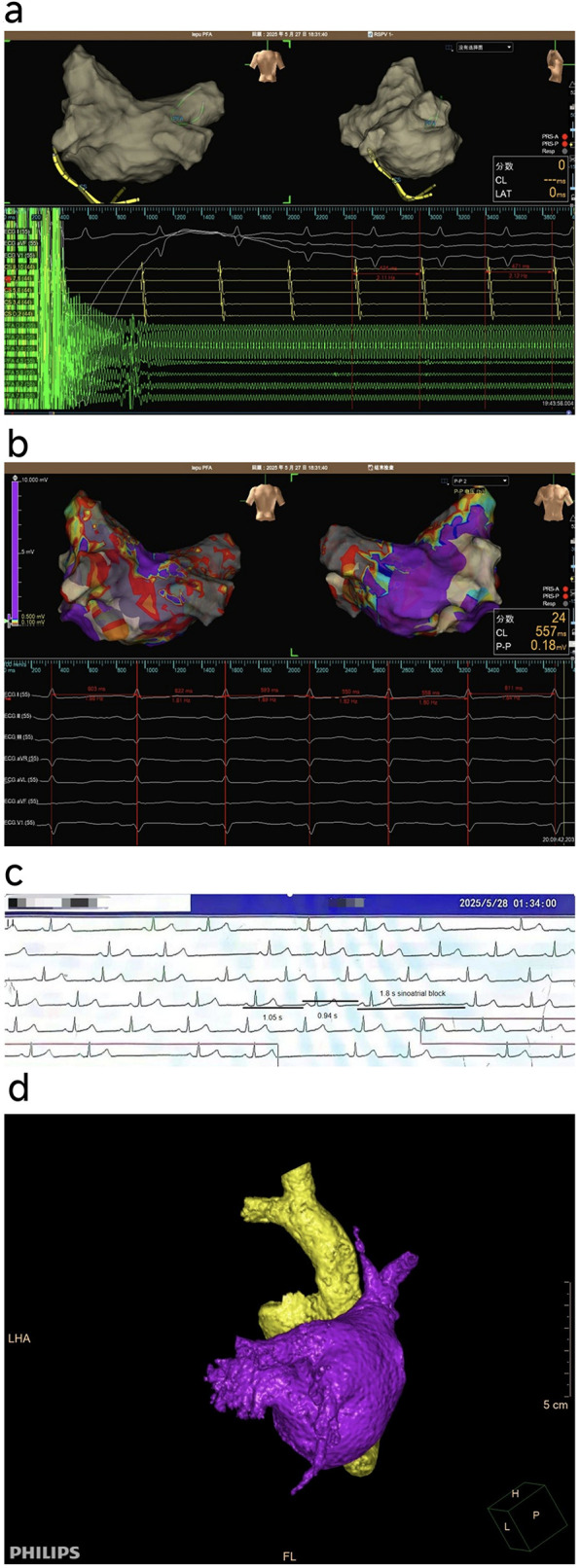
**(a)** Sinus tachycardia during right superior pulmonary vein (RSPV) ablation. **(b)** Normal sinus rhythm and rate observed post-procedure. **(c)** Frequent sinoatrial block observed during postoperative monitoring. **(d)** Bilateral atrial reconstruction showing the relatively large and anteriorly extended RSPV orifice.

A 62-year-old woman with symptomatic paroxysmal AF (burden: 48%) underwent PVI using a circular PFA catheter under general anesthesia. During PFA delivery, key parameters include the pulse count, with each target site typically receiving at least three pulse applications to achieve PVI. The pulse voltage is generally set within a range of 1,500 V to 1,700 V, often starting at 1,500 V inside the pulmonary veins and potentially increasing to 1,600 V or 1,700 V for applications at the ostium or antrum. The catheter configuration features a specialized ablation catheter with two primary deployable shapes: a spindle-shaped configuration for ablation inside the pulmonary veins and a flower-shaped configuration (with six petals) to enhance contact and ablation at the pulmonary vein ostium and antrum regions. Anesthetic drugs include dexmedetomidine (loading dose of 0.5–1.0 μg/kg administered over 10 min before anesthesia induction and followed by a maintenance infusion of 0.2–0.7 μg/kg/h during the procedure) and propofol is administered at a reduced dose - typically 1.0–2.5 mg/kg for induction and 2–6 mg/kg/h for maintenance). During ablation of the right superior pulmonary vein (RSPV), frequent sinus tachycardia (cycle length ≈ 470 ms) was observed (Figures 1Panel a), which was not present during ablation of the other pulmonary veins. This phenomenon has been sporadically observed in other PFA procedures at our center, although it has not been systematically documented. At the end of the procedure, sinus rhythm remained stable, with a cycle length of 550–620 ms (Figures 1Panel b). One hour post-procedure, frequent sinoatrial (SA) block was noted on telemetry (Figures 1Panel c); the patient remained asymptomatic. Without pharmacological intervention, the bradyarrhythmia resolved spontaneously within five hours. No recurrence of AF or pauses exceeding 2 s were observed in Holters on the second day and at one month following the procedure.

Patient consent and ethnic approval (2014-014-01 Tianjin University Chest Hospital) was obtained by participants in this study. They cover all treatments, procedures, and publication of the manuscript with any accompanying images.

## Discussion

The transient SA block observed after RSPV ablation with PFA can be attributed to several interrelated factors, as supported by literature on the electrophysiological effects of PFA and relevant anatomical considerations.

1. Transient SA Nodal Injury Due to PFA: PFA creates large, symmetrical antral lesions through irreversible electroporation, which may transiently affect the SA node when applied in close proximity (2). In this case, the RSPV had a large, anteriorly oriented ostium, positioning it near the SA node region (3). The sinus tachycardia observed during RSPV ablation likely resulted from direct stimulation of the SA node by the electric field, a phenomenon commonly reported in PFA procedures due to its effects on cardiac tissue excitability (4). Subsequently, the delayed SA block may reflect reversible electroporation-induced stunning of the SA node or its microvasculature, similar to cases where PFA caused prolonged but self-resolving asystole without permanent damage (5). PFA lesions exhibit distinctive characteristics compared to radiofrequency ablation, generating larger tissue volumes that may extend unexpectedly beyond the tissue-electrode interface (2, 6). This physical property raises legitimate concerns about potential electrophysiological effects on the SA node, especially when the RSPV orifice of our patient was relatively large and extends forward, which makes it easier for the PFA catheter to approach the SA node (Figures 1Panel d). Extensive clinical experience generally supports the safety of PFA near the SAN, as evidenced by a multicenter study involving 616 cases of SVC isolation, which achieved 100% acute success with minimal transient SAN injury (0.3%) (7). Above are consistent with findings that PFA lesions often exhibit a transient peripheral zone of injury that recovers over hours, explaining the spontaneous resolution observed here (8). Additionally, transient edema resulting from reversible electroporation may contribute to delayed SA node dysfunction by compressing the nodal tissue or its microvasculature, consistent with the self-limiting nature of the observed block. The sinus tachycardia observed during RSPV ablation is likely a direct excitatory effect of the electric field on the SAN, rather than an indication of inadequate analgesia or anesthesia, as it was localized to the RSPV ablation and was not accompanied by other signs of light anesthesia.
2. Autonomic Nervous System Modulation: A study comparing the effects of different ablation methods on the autonomic nerves found that changes in heart rate three months after PFA surgery were negligible compared to those in the radiofrequency ablation and cryoablation groups. Furthermore, when PFA was repeated in the low-voltage area with clearly defined potential isolation, a vagal response could still be induced, suggesting that the effects of PFA on the vagus nerve are reversible (4). Unlike thermal ablation, which commonly induces nerve modulation during pulmonary vein isolation for paroxysmal AF (9–11), PFA does not cause such nerve modulation. Taken together, these findings suggest that the transient dysfunction of the sinoatrial node observed is more likely to have only a temporary impact.
3. Post-anesthesia reaction: Although the patient underwent surgery under general anesthesia, no significant bradycardia was observed during the operation, and the patient had fully regained consciousness before returning to the ward. While residual effects of anesthetic drugs can cause changes in heart rate, in this case, the timing was inconsistent—the bradycardic episode occurred one hour after the operation—and there were no other anesthesia-related symptoms. Moreover, dexmedetomidine's sympathetic inhibition counteracts propofol's parasympathetic suppression, resulting in more stable heart rate, blood pressure, and autonomic balance. This is particularly advantageous during AF ablation, as it minimizes extreme heart rate fluctuations and reduces arrhythmogenic autonomic shifts (12, 13). Therefore, an anesthetic reaction was unlikely to be the primary cause.
4. Potential Thermal Effects of PFA: Although PFA is primarily a non-thermal technology, the high-power pulses can induce some Joule heating in the tissue. Computational models indicate that temperature increases during PFA are generally minimal and unlikely to cause thermal necrosis; however, localized heating near the electrode-tissue interface cannot be entirely excluded (14). In this case, the transient nature of SA node dysfunction and the absence of permanent injury are more consistent with electroporation-mediated effects rather than thermal damage. Nevertheless, further studies are required to clarify whether secondary thermal effects contribute to the observed electrophysiological changes.

This case is novel in demonstrating a temporal sequence of excitatory (tachycardia) followed by inhibitory (SA block) effects after PFA, a phenomenon not widely reported in large series such as MANIFEST-17K (1). It underscores that PFA's effects on the SA node are dynamic and reversible, likely due to its non-thermal mechanism. This pattern contrasts with thermal ablation, where SA node injury is often persistent, and highlights the importance of monitoring for delayed conduction abnormalities following PFA. The successful outcome without recurrence suggests that such transient dysfunction may not require intervention but warrants caution during RSPV ablation.

Since no bradycardia occurred during the operation, we did not perform coronary angiography and therefore could not confirm whether the sinoatrial node artery was involved (15). Additionally, SA node mapping was not conducted to determine the relative distance between the sinoatrial node area and the right upper pulmonary vein.

Identification of Patients at Risk: Based on this case, we recommend using pre-procedural imaging (e.g., cardiac CT) to assess the anatomical relationship between the RSPV and the SAN region. Patients with a large, anteriorly oriented RSPV ostium may be at higher risk for transient SAN dysfunction during PFA. Intraoperative monitoring of sinus rhythm during RSPV ablation is crucial; the appearance of sinus tachycardia may serve as an early warning sign. Prolonged post-procedural monitoring (e.g., telemetry for 24 h) should be considered in such cases.

Recommendations for Monitoring: If sinus tachycardia occurs during RSPV ablation, we recommend continuous telemetry monitoring for at least 24 h post-procedure to detect delayed SA block. Asymptomatic bradyarrhythmias may not require active intervention but should be closely observed. Symptomatic or prolonged pauses may necessitate temporary pacing or pharmacological support.

In conclusion, the transient SA block observed in this patient likely resulted from PFA-induced reversible injury to the SA node, exacerbated by anatomical proximity and autonomic modulation. This case highlights the importance of vigilant post-procedural monitoring following PFA, especially when ablating veins near the SA node, and contributes to the growing body of evidence regarding PFA's unique electrophysiological effects.

## Data Availability

The original contributions presented in the study are included in the article/Supplementary Material, further inquiries can be directed to the corresponding author/s.
