# Supplementary figures and images for: Transient sinoatrial node dysfunction after pulsed-field pulmonary vein ablation: an image case report
Source: Front Cardiovasc Med. 2026 Jan 29;13:1745619. doi: 10.3389/fcvm.2026.1745619 (PMC12894261; doi:10.3389/fcvm.2026.1745619)

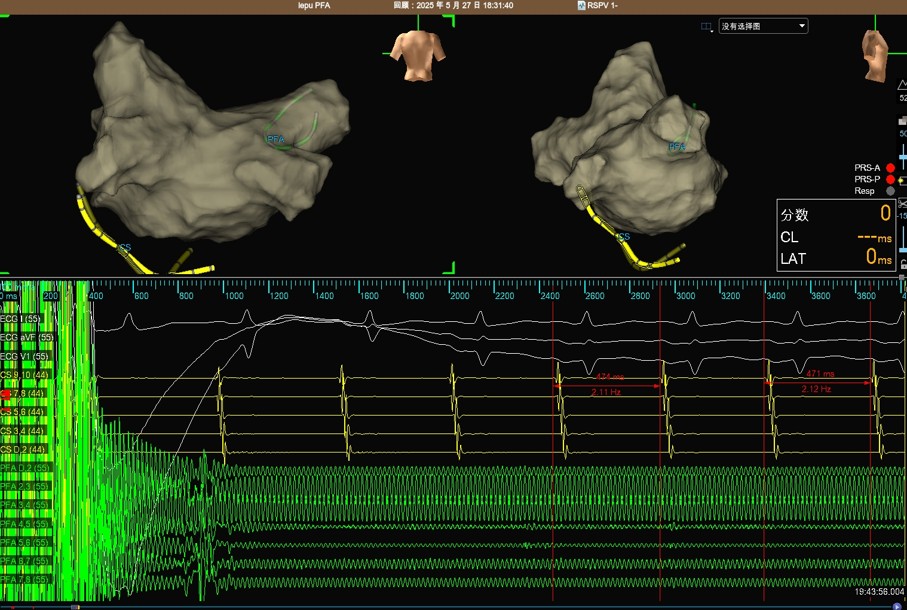

Supplement: Supplementary file 1 [file Image1.jpeg]

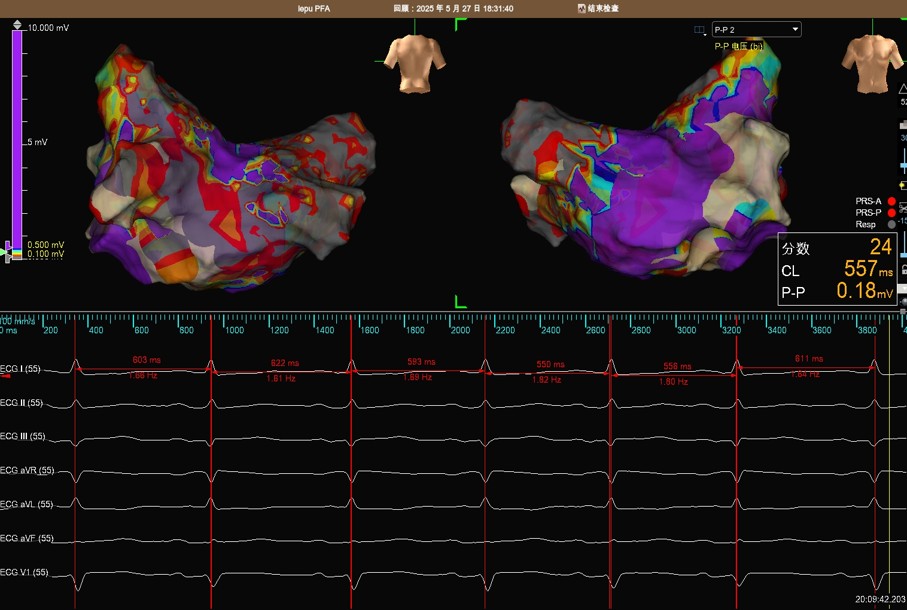

Supplement: Supplementary file 2 [file Image2.jpeg]

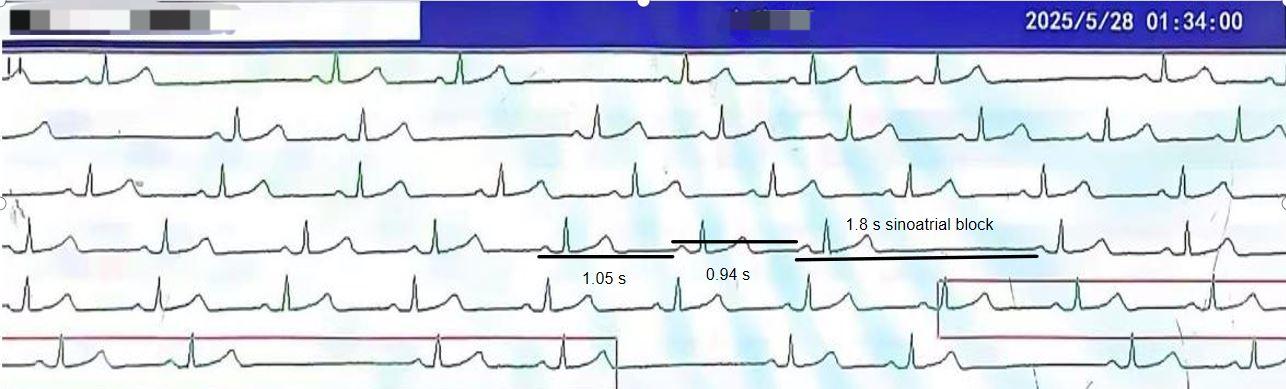

Supplement: Supplementary file 3 [file Image3.jpeg]

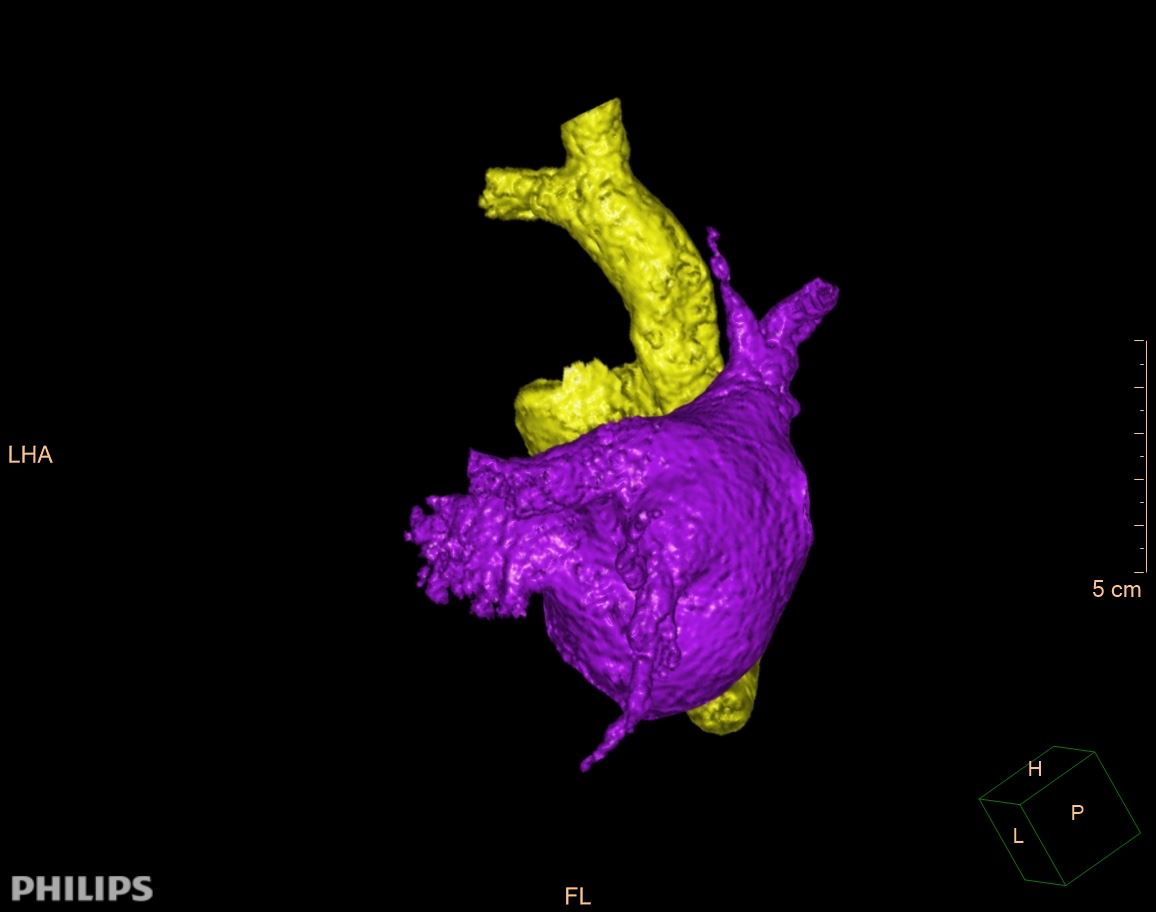

Supplement: Supplementary file 4 [file Image4.jpeg]
